# Case Report: Asymptomatic COVID-19 patient with a subtle hypercoagulable state and fluctuating D-dimer level

**DOI:** 10.12688/f1000research.74009.1

**Published:** 2021-11-03

**Authors:** Jefferson Caesario, Decsa M. Hertanto, Kukuh D. Hernugrahanto, Dwikora N. Utomo, Nicolaas C. Budhiparama, Djoko Santoso, Pancras C.W. Hogendoorn

**Affiliations:** 1 Faculty of Medicine, Airlangga University, Surabaya, 60286, Indonesia; 2 Department of Internal Medicine, Faculty of Medicine, Airlangga University, Surabaya, 60286, Indonesia; 3 Department of Orthopaedic & Traumatology, Faculty of Medicine, Airlangga University, Surabaya, 60286, Indonesia; 4 Nicolaas Institute of Constructive Orthopaedic Research and Education Foundation for Arthroplasty and Sports Medicine, Medistra Hospital, Jakaarta, 12950, Indonesia; 5 Department of Orthopaedic Surgery, Leiden University Medical Center, Leiden, 2333, The Netherlands; 6 Faculty of Vocational Studies, Airlangga University, Surabaya, 60286, Indonesia; 7 Department of Pathology, Leiden University Medical Center, Leiden, 2333, The Netherlands

**Keywords:** COVID-19, Coagulopathy, Asymptomatic, D-dimer, Anticoagulant Therapy

## Abstract

**Background:** COVID-19 can infect an asymptomatic person silently without any overt symptoms despite diffuse blood clots throughout the body. Clot formation is induced by COVID-19 associated coagulopathy that can cause a high mortality rate. D-dimer, a fairly decisive marker for the coagulopathy event, is physiologically a marker of the fibrinolysis process. The increase of D-dimers in COVID-19 cases must be followed up because it relates to the initiation of a cytokine storm.

**Case presentation: **We report an asymptomatic patient with sudden D-dimer elevation who received anticoagulant therapy. After three days of heparin administration, D-dimer results became normal and anticoagulant therapy was stopped. However, on the 12th day, the D-dimer level rebounded back and was followed by an increase of hs-C-reactive protein, erythrocyte sedimentation rate, IL-6, although SARS-CoV-2 PCR result became negative. A hyperglycaemic reaction and a sudden increase of HbA1C was observed in the patient. After three weeks D-dimer had returned to normal levels, and so did the other markers. The patient recovered fully and still no symptoms were obvious.

**Conclusion: **COVID-19 patients without symptoms may be at risk of an asymptomatic coagulopathy process. The decreasing level of D-dimer erroneously cannot ensure that the coagulopathy process stops.

## Introduction

Coronavirus Disease 2019 (COVID-19), following SARS-Cov-2 infection, silently attacks various organs in the human body including the lung. ^1^ Silent hypoxia and blood clots throughout the body are sometimes not followed by any symptoms. ^2^^,^^3^ COVID-19 associated coagulopathy leading to massive thrombosis is the cause of high mortality rates and adds complexity in treating the disease. ^4^^,^^5^ It has been shown to be present in up to 31% of critically ill intensive care unit (ICU) patients. ^5^ COVID-19 associated coagulopathy has a fairly-decisive marker, namely D-dimer, which is a physiological marker of the fibrinolysis process. ^6^ D-dimer elevation in Disseminated Intravascular Coagulation (DIC) generally shows a highly active fibrinolysis process to oppose clot formation. However, the fibrinolysis process in COVID-19 is very minimal when compared to the clot formation. ^7^ This imbalance has the potential to increase mortality. ^8^ Therefore, the increase in D-dimer in COVID-19 cases must be monitored more seriously because it is related to the initiation of a cytokine storm, which causes worsening conditions in COVID-19. D-dimer monitoring is necessary because subclinical thrombosis tends to be missed in asymptomatic cases. ^7^^,^^8^ Here we present an asymptomatic case in which the elevation of D-dimer was present before the cytokine-storm.

## Case report

An Asian male patient, 59-years-old and working as an employee, was found to have a positive result of SARS-CoV-2 virus detection by active tracing after attending a mass event in a closed hall for three hours. The patient had a 15-year history of controlled hypertension, one year of pre-diabetes, and dyslipidaemia, for which he received medical treatment of 16 mg Candesartan, 10 mg Atorvastatin since first diagnosed, and 5 mg bisoprolol after routine medical check-up five years ago. There was a family history of hypertension and diabetes from the patient’s mother, but no psycho-social issues were found.

Periodical Polymerase Chain Reaction (PCR) tests were monitored and COVID-19-positive tests were found for two weeks while no symptoms were felt by the patient ( Table 1). During the two weeks, a complete exploration of laboratory results such as hematological test, liver function, renal function, and urinalysis was observed, including markers of coagulopathy and cytokine-storm.

**Table 1.** COVID-19 PCR results for the patient over four weeks.

Date	PCR result
28/12/20	Negative
04/01/21	Positive
07/01/21	Positive
11/01/21	Positive
18/01/21	Negative
22/01/21	Negative

Since dealing with a very unpredictable disease, once the original PCR result was obtained, aggressive therapy, as stipulated by previous studies, ^9^ was given immediately. The therapy, received by the patient at a private clinic, included 5 g intravenous immune globulin for three days and 10 g for the next three days (with premedication of 5 mg dexamethasone intravenous for six days), 2 g intravenous meropenem for six days, and 2400 mg favipiravir (first day) and 1200 mg for ten days.

**Figure 1.**
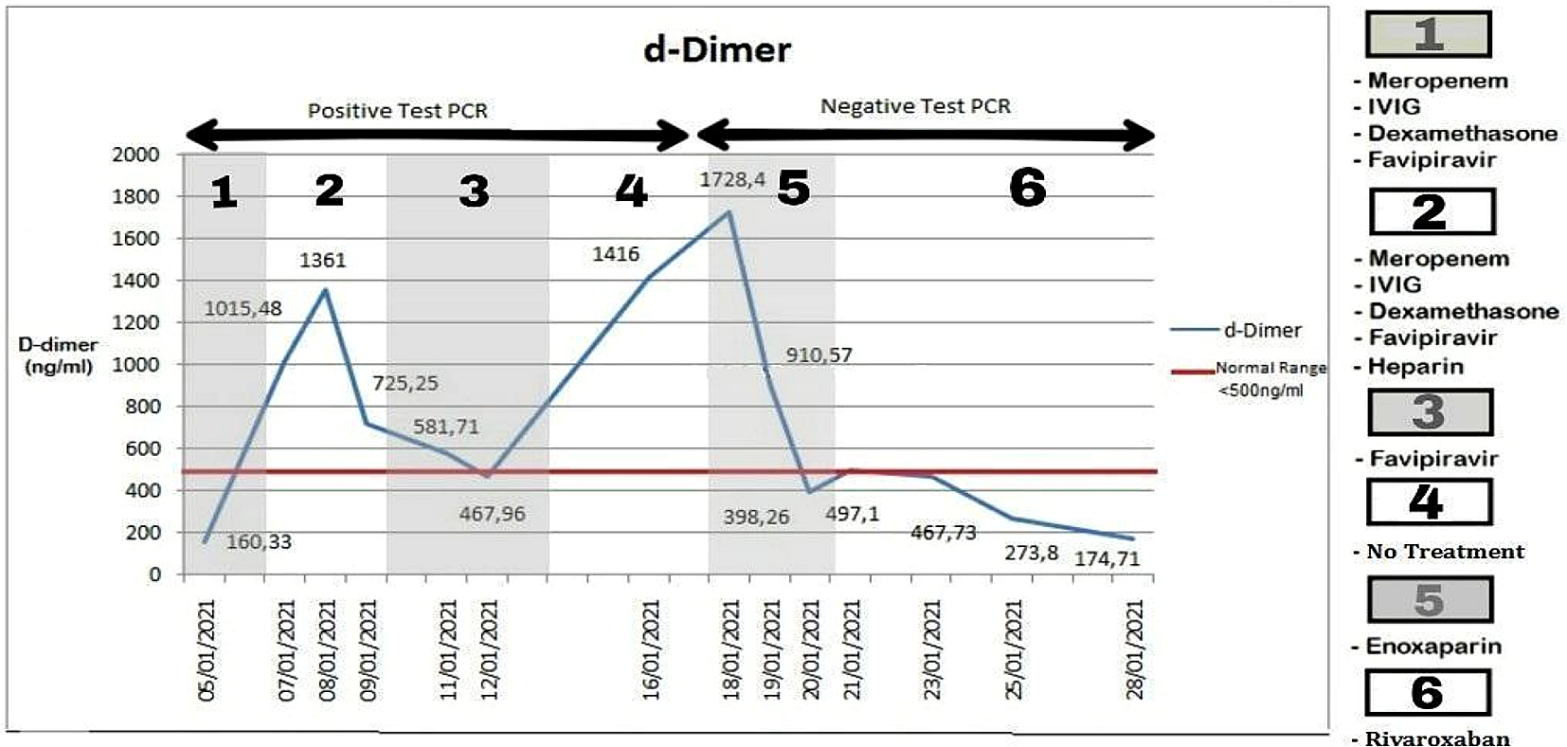
D-dimer levels of the patient over three weeks. Note: bold numbers including those in boxes indicate batches of therapy regimens.

On the third day, anticoagulant heparin (9000 IU) was given since there was a sudden spike of D-dimer from 160 to 1015 ng/mL in a day that was beyond the normal value (<500 ng/mL) ( Figure 1). After three days of heparin administration, on the seventh day, the D-dimer result decreased to 467 ng/mL and heparin was stopped. However, after four days, on the 12th day D-dimer level monitoring showed a second spike of 1416 ng/mL and was followed up by 1728 ng/mL two days after. Enoxaparin (6000 IU) was given for three days, and anticoagulant treatment continued with rivaroxaban 10 mg for three weeks. Eventually, after three days, D-dimer results showed continuously normal levels below 500 ng/mL. The findings, however, became a particular concern since the D-dimer second spike coincided with the conversion of the COVID-19 PCR result to negative.

**Figure 2.**
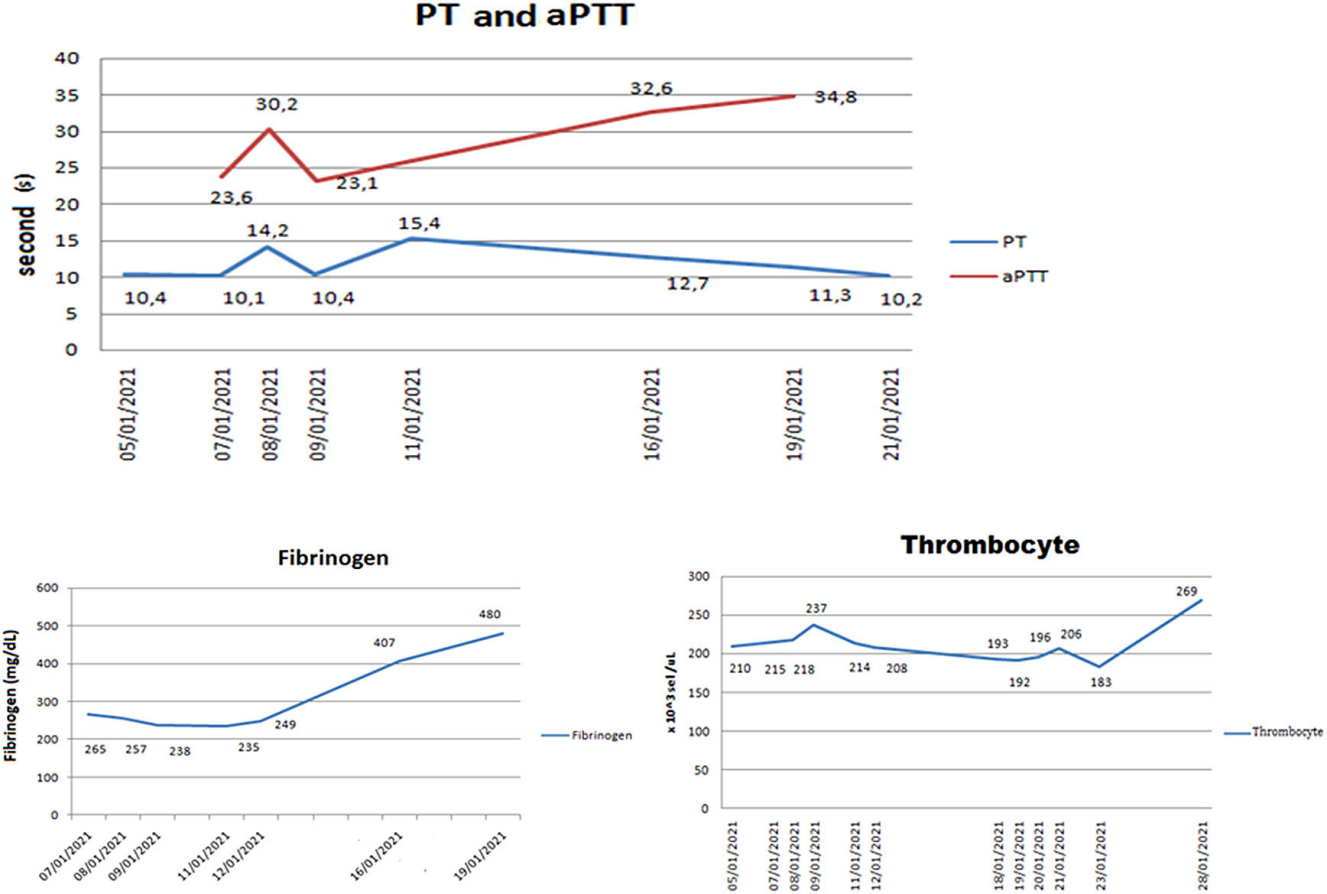
Partial Thromboplastin Time (aPTT), Prothrombin Time (PT), Fibrinogen, and Thrombocyte levels of the patient.

In general, D-dimer elevation is closely associated with DIC so other laboratory tests such as Activated Partial Thromboplastin Time (aPTT), Prothrombin Time (PT), fibrinogen, and platelets were performed from the first day of treatment for COVID-19. These markers showed unremarkable changes. The patient showed normal or slightly elevated levels of aPTT and PT in the range of 23.1-34.8 s and 10.1-15.4 s, respectively (normal value: 23.0-30.2 s for APTT and 10.1-11.9 s for PT) ( Figure 2). The PT level increase was still less than 3 seconds, so the elevation had not fulfilled the criteria as in guidelines of The International Society for Thrombosis and Haemostasis (ISTH). ^10^

Since DIC is also associated with excessive use of fibrinogens and platelets for clot formation, in this patient its levels should have declined. Fibrinogen levels of the patient (235-480 mg/dL) were found in or with a slight increase to the normal range (200-400 mg/dL). According to the ISTH classification, the decrease of fibrinogen in this case, is insignificant, unless <100 mg/dL. Platelet levels of the patient (183,000-269,000 cells/uL) were also in the normal range (150-450 × 10 ^3^ cells/uL), whereas significant platelet levels, according to the ISTH criteria, are <100,000 cells/uL.

**Figure 3.**
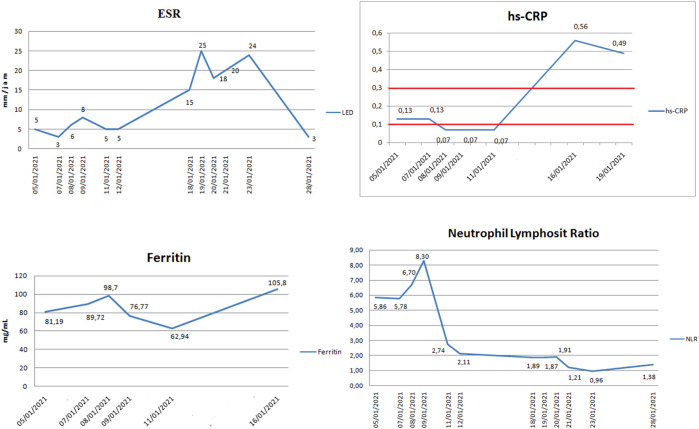
Erythrocyte sedimentation rate (ESR), C-reactive protein (CRP), Ferritin, and Neutrophil-Lymphocyte Ratio (NLR) of the patients.

Markers of a cytokine-storm were observed, such as Interleukin-6 (IL-6), Ferritin, Erythrocyte Sedimentation Rate (ESR), and C-Reactive Protein (CRP). The Neutrophil-Lymphocyte Ratio (NLR) was also examined to predict the progression of COVID-19. When D-dimer increased for the second time, there was a surge of hs-CRP and ESR ( Figure 3) of 0.56 mg/dL and 15-25 mm/hour, respectively, which were above the normal range of <0.5 mg/dL and < 10 mm/hour, respectively. IL-6 examination was carried out three times and showed an increased level of 5.52-10.85 pg/mL above normal level (<7 pg/mL) ( Table 2). On the contrary, the patient's ferritin level increased to normal levels while the NLR was relatively stable. These unique findings strongly displayed the abnormalities in laboratory results on the 12th day, tending to a profile of cytokine storms.

**Table 2.** IL-6 results for the patient over four weeks.

Date	IL-6 (pg/mL)
08/01/2021	<1.5
16/01/2021	5.52
05/02/2021	10.85

**Figure 4.**
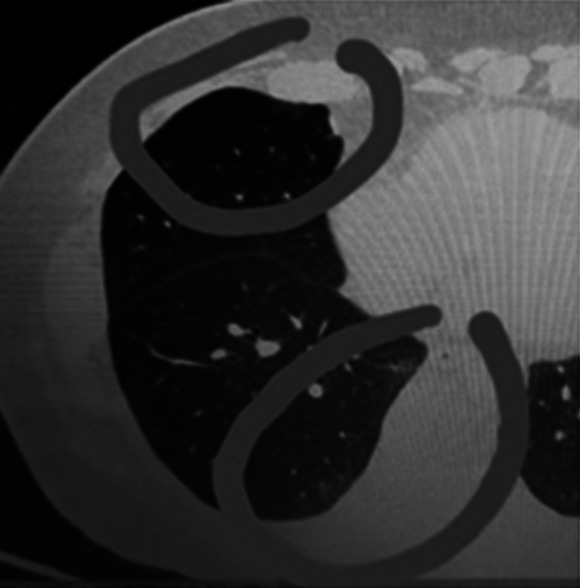
CT-scan chest/lung with 128 slices of the patient on the 12th day of treatment. Circles indicate ground-glass appearance on the right lobe lung.

In addition to laboratory results, lung radiological examination and chest X-rays were performed and showed normal results on the 1st, 7th, and 12th days. On the 12th day, when D-dimer increased for the second time, accompanied by an increase in other inflammatory markers, the patient underwent a chest CT scan, which showed a minimal ground glass appearance in the right lobe of the lung ( Figure 4).

**Figure 5.**
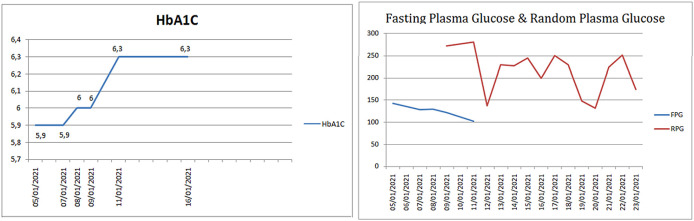
HbA1C, fasting plasma glucose, and random plasma glucose of the patient.

Other laboratory examinations were examined to rule out the causes of increased D-dimers due to non-COVID-19 causes, such as autoimmune, endocrine disorders, and malignancy. Complement proteins (C3 and C4), free-T4, T3, and TSH, Alpha-Fetoprotein (AFP), Carbohydrate Antigen 19-9 (CA 19-9), and Carcinoembryonic Antigen (CEA) were all normal. The patient's blood glucose profile was also observed due to a history of prediabetes. HbA1C level increased from 5.9% to 6.3% within one week. The data of random plasma glucose increased inconsistently despite insulin administration ( Figure 5).

After four weeks of treatment, the patient fully recovered and still did not feel any symptoms of COVID-19 and completed four weeks of rivaroxaban.

## Discussion

COVID-19 has two silent killer mechanisms: silent hypoxia and massive thromboembolism, where a considerable number of SARS-CoV-2 infected individuals can be asymptomatic. ^1^^–^^3^ Silent hypoxia can be detected using pulse oximetry. Post-mortem reports indicate that thromboembolism is a more complex process involving a complicated interaction of SARS-CoV-2 virus invasion with platelets, endothelial cells, leukocytes, inflammation, and immune response. Reports revealed that 59.6% of severe COVID-19 cases showed an increase in D-dimer level (threshold as 0.5 mg/L), decreased platelet count (thrombocytopenia), and prolonged prothrombin time. ^11^

A thrombo-embolism event in an asymptomatic individual frequently happens and can be fatal. ^9^^,^^12^ Cui *et al* investigated thrombosis events that occurred in asymptomatic ICU patients using ultrasonography imaging showing an incidence of 25% (20/81). ^13^ Del Nonno *et al* also discovered the presence of a large thrombus clogging the bifurcation of the main pulmonary artery in autopsy despite asymptomatic history. The asymptomatic thrombosis process led to sudden death and surprisingly can possibly occur after the swab results becoming negative. ^12^ Moreover, pulmonary thromboembolism should be considered as the cause of the sudden onset of oxygenation degradation and hypoxia. ^10^^,^^12^

Our case highlighted D-dimer's importance as a screening tool for a thromboembolism event since D-dimer could indicate COVID-19 associated coagulopathy. ^6^ The D-dimer elevation of patients infected by SARS-CoV-2 is strongly related to poor prognostic results and the need for intensive care management. ^5^^,^^8^^,^^14^ Poor prognosis included patients with mild and no symptoms. ^12^ D-dimer early spike detection, related to COVID-19, can signal the need for additional treatments such as anticoagulant therapy before the coagulopathy process develops massively. ^7^^,^^8^

In general, such as in DIC-sepsis cases, D-dimer shows fibrinolysis’ escalation to balance the clotting process that occurs throughout the body. ^7^ On the contrary, in COVID-19 coagulopathy, the fibrinolysis process remains inefficiently 0.02% times its normal capability to encounter clotting formation. ^7^^,^^8^ Due to that unique characteristic of coagulopathy, physicians should treat their patients carefully since a small D-dimer elevation above 500 ng/mL could represent a severe clotting process that occurs quickly and demands immediate treatment. If this condition is not handled correctly, patients can suddenly fall into a fatal hypercoagulability state. ^8^^,^^15^ The administration of anticoagulant agents, such as enoxaparin, heparin, and apixaban, demonstrate a significant reduction in the mortality rate of COVID-19 coagulopathy. ^16^

In this case report, the patient showed relatively normal values of fibrinogen, platelet, APTT, and PT which was different from common DIC related to sepsis. APTT prolongation, fibrinogen depletion, and platelet deprivation can be found in COVID-19 in the later stage due to sepsis. ^7^ However, in contrast, in this case, normal values of the markers were probably due to the therapeutic management applied to the patient.

PCR tests for this patient had been negative on day 15, but on day 12 until 15 the patient experienced a D-dimer rebound after discontinuing anticoagulation therapy, which happened simultaneously with the elevation of ESR, CRP, and IL-6 as cytokine storm markers. Studies showed D-dimer elevation appeared approximately five days before the cytokine storm and probably induced the cytokine storm. ^10^ This case showed that anticoagulant discontinuation after normal D-dimer value could lead to a misjudgement that affects the therapeutic result.

COVID-19 and hyperglycaemia are clinical problems. According to the CORONADO study (Coronavirus SARS-CoV-2 and Diabetes Outcomes), people with diabetes that have a hypoglycaemic condition at the time of hospital admission seem to worsen the prognosis of COVID-19. ^17^ People without diabetes but having hyperglycaemia also have a poorer prognosis of COVID-19 than people with diabetes. ^18^ HbA1c is also associated with inflammation, hypercoagulability, low SaO _2_, and mortality in COVID-19 patients. Early determination of HbA1c in hospital admission can help assess inflammation, hypercoagulability, and prognosis of COVID-19 patients. ^19^ Initial therapy to lower the hyperglycaemic conditions is essential, besides management of infection, inflammation, and supportive care. This management can prevent a prolonged poor condition and subsequent poor prognosis. ^18^

Those clinical manifestations were undoubtedly related to our patient's medical history of hypertension and prediabetes, which affected the coagulation process's quality. The sudden changes of HbA1C and the hyperglycaemic reaction showed unusual findings since erythrocytes survive for three months. ^19^^,^^20^ These blood glucose impairments do not seem related to the thromboembolism process. ^20^

Another note from this case was that the progression to cytokine storm could happen even after the conversion of PCR to negative result. ^12^ If the management of COVID-19 is only focused on the PCR result, then the coagulopathy and cytokine storm can be missed by clinicians, which may lead to a false assumption that the cause of death is other factors, instead of COVID-19. A post-COVID-19 physical and laboratory follow-up is propagated to objectify the recovery of COVID-19 infection patients, especially focussing on functional impairment. ^20^ Within this scale, our patient would have fitted in the grade 0 cohort. It would be very interesting to have long term follow-up of a cohort of these grade 0 patients.

## Conclusion

When facing COVID-19, with its diverse clinical spectrum, clinicians should be cautious. Coagulopathy associated with COVID-19 can occur without any symptoms but probably leads to worse results. D-dimer can become a reliable screening tool against COVID-19 thromboembolism to anticipate the asymptomatic presence of COVID-19 to provide early necessary treatments for preventing fatality.

## Consent

Written informed consent for the publication of this case report and any associated images was obtained from the patient.

## Data availability

All data underlying the results are available as part of the article and no additional source data are required.
