# Supplementary material for: Case Report: Asymptomatic COVID-19 patient with a subtle hypercoagulable state and fluctuating D-dimer level
Source: F1000Res. 2021 Nov 3;10:1112. [Version 1] doi: 10.12688/f1000research.74009.1 (PMC8861469; doi:10.12688/f1000research.74009.1)
Supplement: Peer review history

# Peer review: Reviewer response for version 1

**DOI:** 10.5256/f1000research.77714.r121288

**Published:** 2022-02-21

**Authors:** Najib Alwi

**Affiliations:** 1 Faculty of Medicine, Management and Science University, Shah Alam, Malaysia

This Case Report has been clearly written, highlighting the otherwise possibly missed rise in D-dimer level in asymptomatic Covid-19 positive patients. The main strength of the report is a comprehensive workout of the patient, including all relevant lab and radiological investigations to support the issue that is being highlighted. The case has also been sufficiently discussed, including comparing the D-dimer elevation phenomena with DIC. Strongly supported for acceptance for indexing.

Are enough details provided of any physical examination and diagnostic tests, treatment given and outcomes?

Yes

Is the case presented with sufficient detail to be useful for other practitioners?

Yes

Is sufficient discussion included of the importance of the findings and their relevance to future understanding of disease processes, diagnosis or treatment?

Yes

Is the background of the case’s history and progression described in sufficient detail?

Yes

Reviewer Expertise:

Neuroscience, mental health, impacts of Covid-19 on society.

I confirm that I have read this submission and believe that I have an appropriate level of expertise to confirm that it is of an acceptable scientific standard.

# Peer review: Reviewer response for version 1

**DOI:** 10.5256/f1000research.77714.r99043

**Published:** 2021-11-29

**Authors:** Bayu Satria Wiratama

**Affiliations:** 1 Graduate Institute of Injury Prevention and Control, College of Public Health, Taipei Medical University, Taipei, Taiwan

This study provided a useful insight into the clinical characteristics of thromboembolism risk on asymptomatic patients. There are few questions that need to be answered about this study:

1. In figure 2, it will be more informative if the authors added the D-dimer level on each of the figures to give more useful information.
2. In this study, please use consistent date information, for example if authors use day 1, day 12 then in the chart please stick to the same information. If authors use exact date such as 16/01/2021 then please use the same one in the text.
3. In the last sentence of Introduction, authors mentioned that ".. the elevation of D-dimer was present before the cytokine-storm". It would be interesting if authors could elaborate and describe more using the clinical data about D-dimer elevation and cytokine storm. For example, in figure 3, authors could try adding D-dimer inside each charts about cytokine storm parameters.

Are enough details provided of any physical examination and diagnostic tests, treatment given and outcomes?

Partly

Is the case presented with sufficient detail to be useful for other practitioners?

Yes

Is sufficient discussion included of the importance of the findings and their relevance to future understanding of disease processes, diagnosis or treatment?

Yes

Is the background of the case’s history and progression described in sufficient detail?

Yes

Reviewer Expertise:

epidemiology, injury, COVID-19

I confirm that I have read this submission and believe that I have an appropriate level of expertise to confirm that it is of an acceptable scientific standard.
